# Supplementary material for: Multiplex imaging relates quantal glutamate release to presynaptic Ca2+ homeostasis at multiple synapses in situ
Source: Nat Commun. 2019 Mar 29;10:1414. doi: 10.1038/s41467-019-09216-8 (PMC6441074; doi:10.1038/s41467-019-09216-8)
Supplement: Supplementary file 1 — Supplementary Information [file 41467_2019_9216_MOESM1_ESM.pdf]

Multiplex imaging relates quantal glutamate release to presynaptic Ca^{2+} homeostasis at multiple synapses *in situ*

by Jensen *et al.*

Supplementary Information

Supplementary Figures

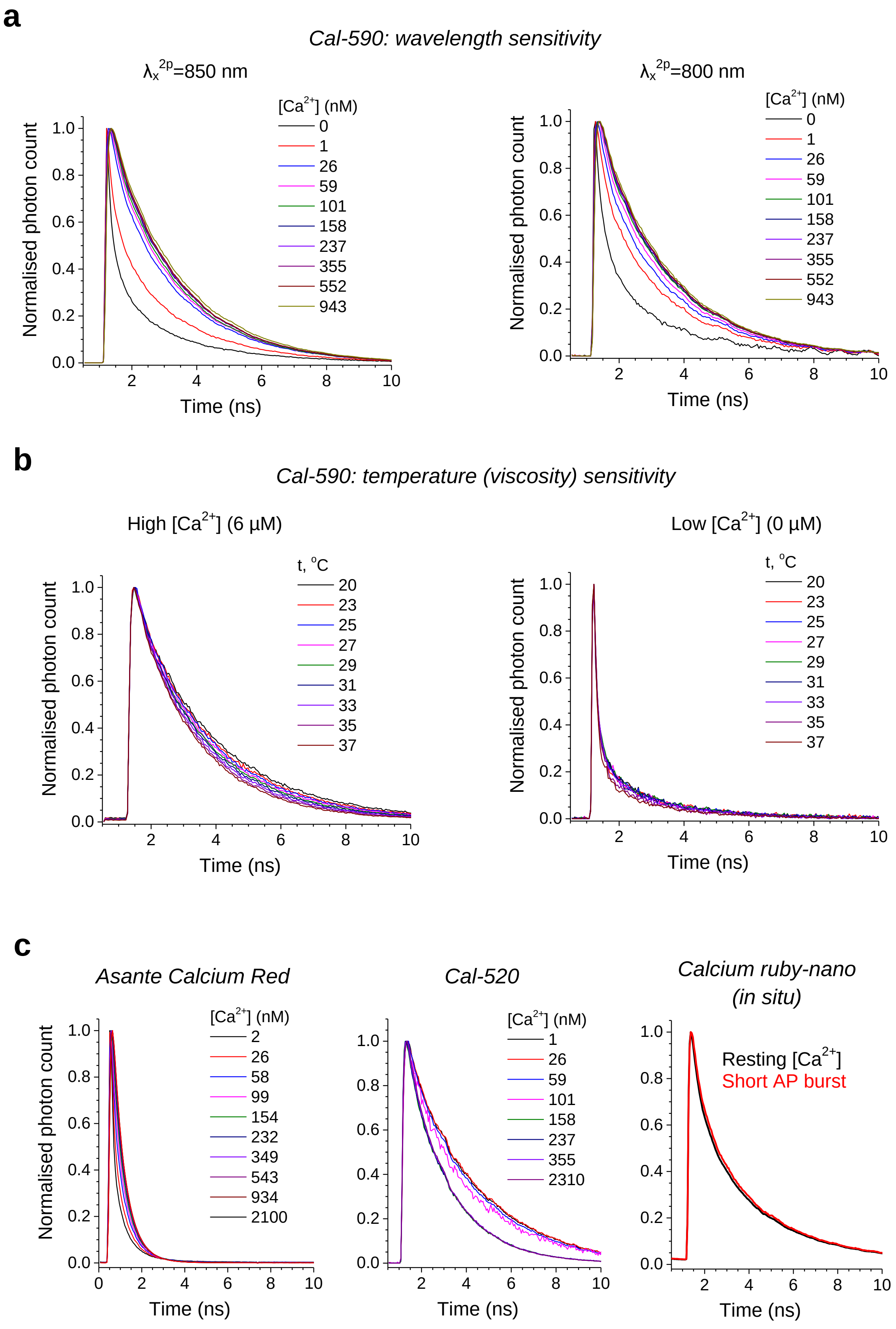

Supplementary Figure 1. Cal-590 fluorescence lifetime sensitivity to excitation wavelength and temperature / viscosity.

(a) Cal-590 lifetime sensitivity to [Ca^{2+}] under different two-photon excitation wavelength, 850 nm and 800 nm, as indicated (temperature 33ºC). The detected sensitivity range for 0-200 nM [Ca^{2+}] is narrower than that for the optimal excitation wavelength of 910 nm (Fig. 1a-b).

(b) Fluorescence decay of Cal-590 under saturating (left) and zero-clamped (right) [Ca^{2+}], over the range of experimentally relevant temperatures, as indicated (viscosity).

(c) Testing the fluorescence decay [Ca^{2+}] sensitivity for Asante Calcium Red, Cal-520, and ruby-nano, as indicated: the first two were evaluated using the standard FLIM calibration procedure as in **a-b** whereas Calcium ruby-nano was tested *in situ* (axonal bouton, CA3 pyramidal cell, organotypic hippocampal slices), by comparing its fluorescence decay in resting conditions (black) and during peak intensity response to a burst of four action potentials (at 20 Hz, red), as indicated; recordings at λ_x^{2p} = 910 nm, 33ºC.

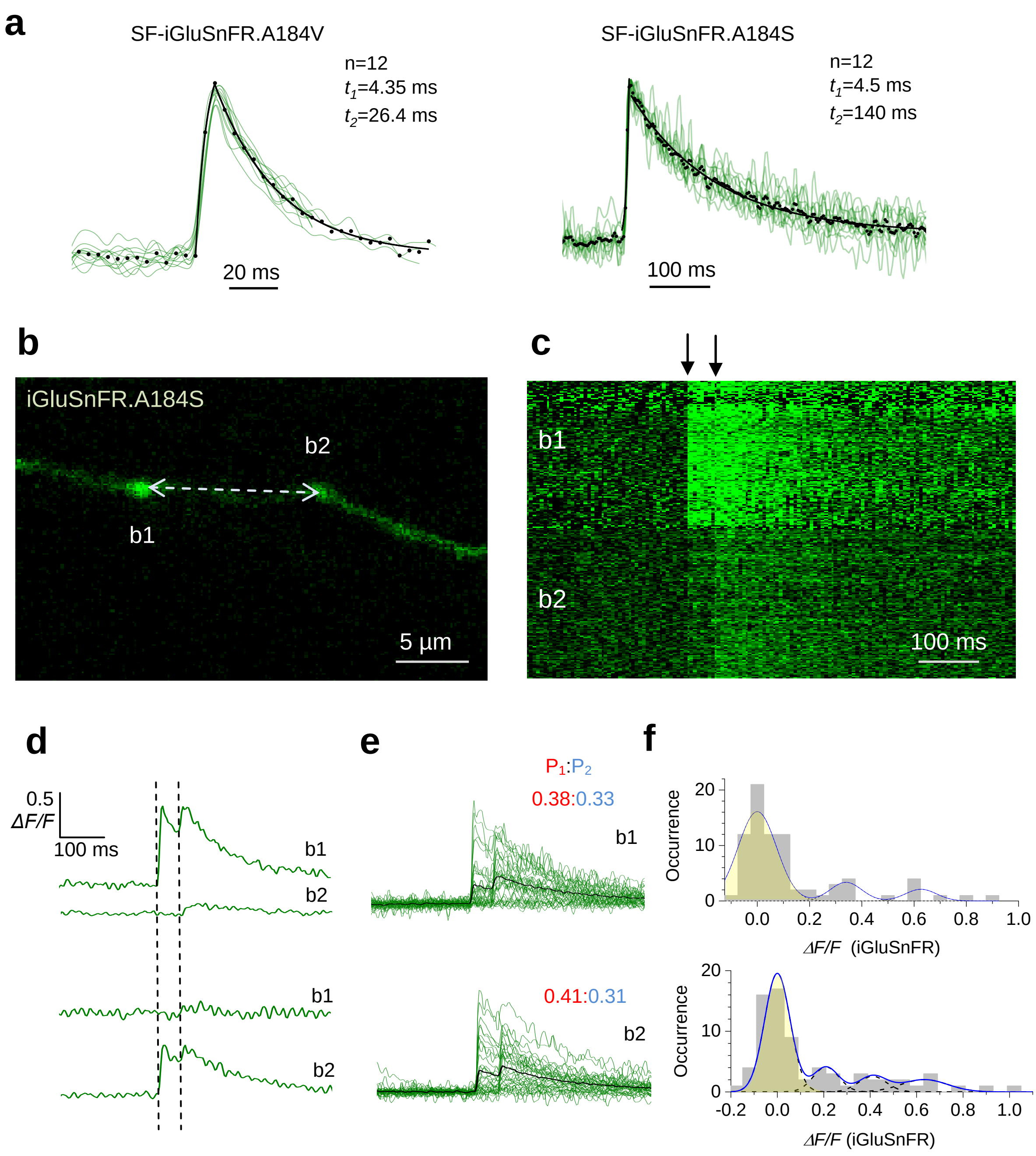

Supplementary Figure 2. Simultaneous multi-synapse imaging of quantal glutamate release with iGluSnFR.A184S.

(a) Comparative assessment of the binding (fluorescence) kinetics for SF-iGluSnFR.A184V and SF-iGluSnFR.A184S variants, as indicated, upon quasi-instantaneous glutamate release; green traces, normalised single-AP-evoked fluorescence responses recorded in individual boutons of CA3 pyramidal cell axons traced to area CA1; dots, all-trace average; black line, best-fit double-exponent approximation: t_1, rise constant; t_2, decay constant; n, number of trials.

(b) CA3 pyramidal cell axon fragment in area CA1 showing two presynaptic boutons (b1-b2); two-headed arrow illustrates laser scan trajectory, with the scanning dwell points in the bouton centres.

(c) A pseudo-linescan image of SF-iGluSnFR.A184S signals recorded simultaneously (one sweep example) at two boutons shown in **a** as indicated, during somatic generation of two APs 50 ms apart (arrows); glutamate releases and failures can be seen; further detail in Fig 2b.

(d) Example of two consecutive recordings (top and bottom; *ΔF/F* SF-iGluSnFR.A184S) from two boutons, as indicated, to illustrate no fluorescence signal cross-talk between the boutons. P1:P2, average probability of the first (red) and second (blue) release events.

(e) A summary of 36 trials (1 min interval) in the experiment shown in **a-b**; green traces, single-sweep SF-iGluSnFR.184S *ΔF/F* intensity readout at the two bouton centres; black traces, all-sweep-average.

(f) Amplitude histograms (SF-iGluSnFR.184S *ΔF/F* signal, first and second response counts combined; pre-pulse 8 ms baseline subtracted), with a semi-unconstrained multi-Gaussian fit (blue line, Methods) indicating peaks that correspond to estimated quantal amplitudes; the leftmost peak corresponds to zero-signal (failure; yellow shade); dotted lines, individual Gaussians; arrows, average amplitudes (including failures) of the first (red) and second (blue) glutamate response.

a

b

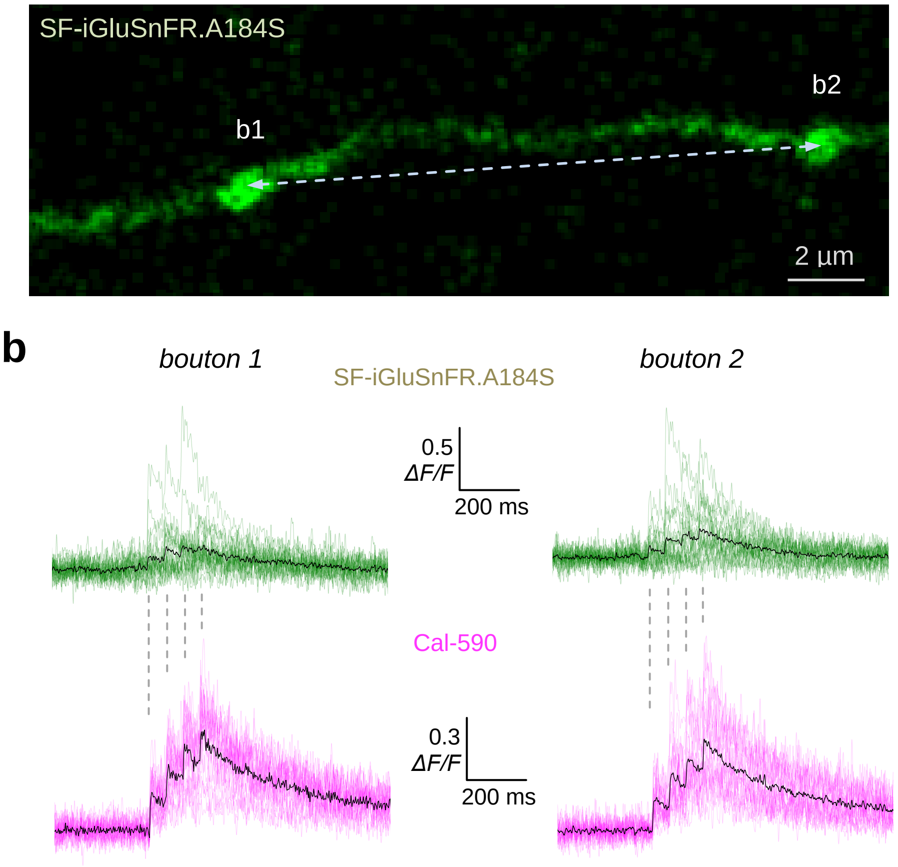

Supplementary Figure 3. Simultaneous multi-synapse imaging of quantal glutamate release and presynaptic Ca^{2+} dynamics with SF-iGluSnFR.A184S and Cal-590.

(a) CA3 pyramidal cell axon fragment in area CA1 showing two presynaptic boutons (b1-b2; green channel); two-headed arrow illustrates laser scan trajectory, with the scanning dwell points in the bouton centres.

(b) A summary of 24 trials (1 min interval) in the experiment shown in **a**; green and magenta traces, single-sweep SF-iGluSnFR.A184S and Cal-590 *ΔF/F* intensity readout at the two bouton centres; black traces, all-sweep-average.

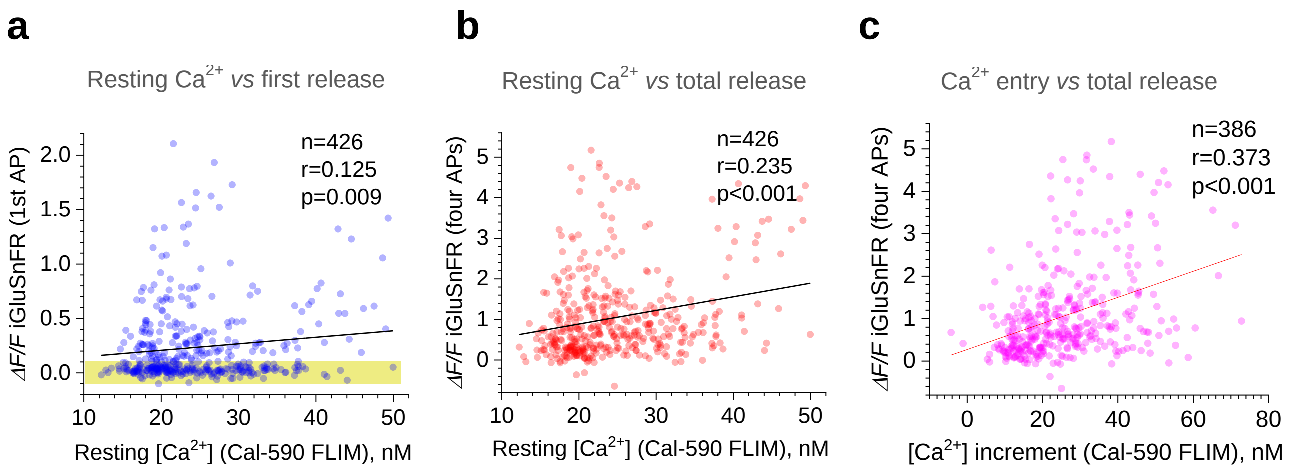

Supplementary Figure 4. Resting presynaptic Ca^{2+} and evoked Ca^{2+} entry both control glutamate release reported by direct SF-iGluSnFR.A184V readout.

(a) Glutamate release (*ΔF/F* SF-iGluSnFR.A184V signal) upon first AP, plotted against resting [Ca^{2+}] (Cal-590 FLIM readout, averaged over 100 ms pre-pulse). Solid line, linear regression (r, Pearson's correlation; p, regression slope significance; n, number of events, N = 26 axonal boutons recorded); yellow shade, Gaussian noise (failure response) cut-off, ~95% confidence interval.

(b) Cumulative glutamate release upon four APs (*ΔF/F* iGluSnFR signals summed, with pre-AP 8 ms baselines subtracted) plotted against presynaptic resting [Ca^{2+}] (Cal-590 FLIM readout); other notations as in a.

(c) Cumulative glutamate release upon four APs (as in c), plotted against cumulative [Ca^{2+}] increment (Cal-590 FLIM readout, during four APs); other notations as in a (N= 24 boutons recorded).

See Fig. 4b-d for the corresponding data reporting quantal content of glutamate release.

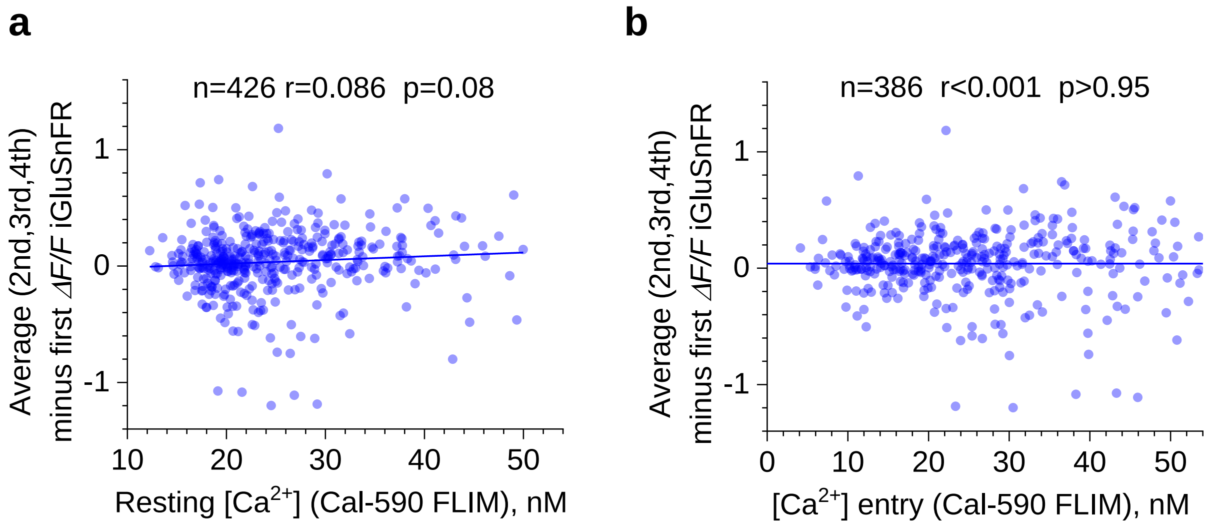

Supplementary Figure 5. Resting presynaptic Ca^{2+} and evoked Ca^{2+} entry are not correlated with short-term plasticity (facilitation or depression) of glutamate release.

(a) Short-term plasticity indicator calculated as the average amplitude of the 2nd, 3rd, and 4th *ΔF/F* iGluSnFR signals minus the amplitude of the 1st *ΔF/F* iGluSnFR signal (formula to minimise fluctuations due to release failures) plotted against presynaptic resting $[Ca^{2+}]$ (Cal-590 FLIM readout).

(b) Short-term plasticity indicator as in **a** plotted against cumulative $[Ca^{2+}]$ increment (Cal-590 FLIM readout, during four APs); trials recorded with only two (rather than four) APs were excluded; other notations as in **a**.

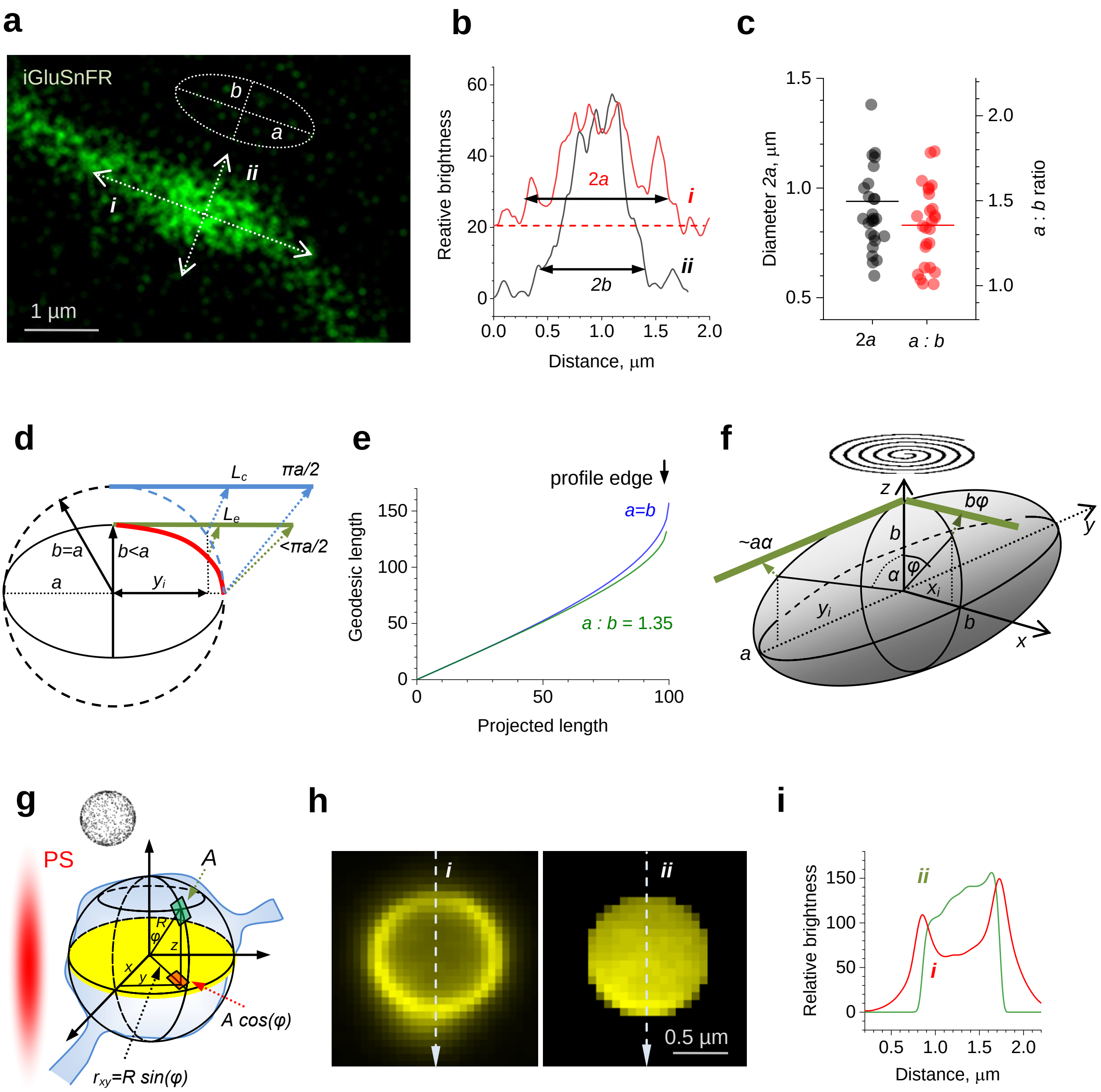

Supplementary Figure 6. Stereological geodesic corrections for the signal spread and density on spherical or elliptical surfaces (of axonal boutons) projected onto the focal plane.

(a) An axonal bouton (example) projection in the focal plane, with two profile sampling lines (*i* and *ii*) to estimate outline approximation by an ellipse (dotted oval), with *a* and *b* being major and minor axes, respectively.

(b) Brightness profiles along lines *i* and *ii* shown in **a**, as indicated. Two-headed arrows, effective width of the bouton along *i* and *ii* axes, to provide *a* and *b* values for the approximating ellipse, as shown. The cut-off values for *a* and *b* (arrow end

positions) correspond to ~2SD of the background noise; horizontal dotted line, background reflecting the adjacent axon.

(c) Data scatters showing *a* values (mean ± SEM, 0.59 ± 0.02) and *a* : *b* ratios (1.35 ± 0.04) in the recorded sample of boutons (n = 26).

(d) Trigonometry diagram explaining geodesic corrections for distances on curved surfaces that are projected onto the (focal) *x-y* plane (as in Fig. 5d). For an elliptical section with major and minor axes *a* and *b*, respectively, the projected distance x_i from the centre corresponds to the geodesic distance (L_e, green segment) which is consistently larger than that for a circular correction (L_c, blue segment).

(e) Comparison between L_e and L_c geodesic corrections (relative 1-100 scale), with the average *a* : *b* experimental ratio of 1.35 (as in **c**). The discrepancy between the two is small throughout the length reaching a maximum of ~15% towards the projection edge (elongated tip of the ellipsoid in **d**).

(f) Summary schematic for geodesic correction applied to a rotational ellipsoid (approximating the axonal bouton shape) projected onto a plane. For the running coordinate x_i, the corrected geodesic distance (along the *x* axis) is $b\varphi = b$ arcsin (x_i / b) whereas for the coordinate y_i, the geodesic distance (along the *y* axis) is ~ $a\alpha = a$ arcsin (y_i / a). Because the tornado scan (shown) is normally a circle inscribed into the bouton oval (Fig. 5e), there will be no data collected towards the tip of the ellipse, in the *y* direction (Fig. 5f). Thus, the ~15% discrepancy between circular and elliptic corrections which is seen towards the profile edge in the *y* direction (seen in **e**) will be effectively void, ensuring good approximation (estimated error <5%).

(g) Diagram illustrating stereological correction for the surface signal density on a spherical surface; inset, cartoon showing that the planar projection of spherical objects, with uniform surface distribution of fluorescence signals (dots), yields overestimated signal density towards the projection edges; red shade, approximate representation of the microscope's point-spread function (PSF). Sphere-like bouton structure is shown projecting onto the microscope focal plane (yellow)**.** In spherical coordinates (indicated), the differential (infinitesimal) element *A* of the sphere surface (green quadrangle) has an area of $r^2 \cdot sin(\varphi) d\varphi d\theta$ whereas its projection onto the *z*=0 plane (red quadrangle) has an area of $r^2 \cdot sin(\varphi) \cdot cos(\varphi)\ d\varphi d\theta$. The signal intensity in the projection is therefore boosted by factor $1 / cos(\varphi)$ compared to the surface intensity. Thus, applying the factor $cos(\varphi) = (1 - sin^2(\varphi))^{1/2} = (1 - r_{xy}^2 / r^2)^{1/2}$ to the image intensity provides the projection-corrected value.

(h) *Left,* focal plane projection of a nanoengineered microcapsule containing a fluorescent dye in its shell only (left) [1]. *Right*, the capsule shell fluorescence map corrected for the stereological bias as explained in **g**; dotted lines (*i, ii*), brightness profile sampling.

(i) Brightness profiles sampled in images shown in **h**, as indicated.

Supplementary Reference

1 Kopach, O. *et al.* Nano-engineered microcapsules boost the treatment of persistent pain. *Drug Deliv* 25, 435-447 (2018).
